# Retraction Note: microRNA-29b prevents renal fibrosis by attenuating renal tubular epithelial cell–mesenchymal transition through targeting the PI3K/AKT pathway

**DOI:** 10.1007/s11255-025-04664-2

**Published:** 2025-07-10

**Authors:** Shuang Hu, Hongtao Hu, Rui Wang, Hong He, Hua Shui

**Affiliations:** 1 Department of Nephrology, Zhongnan Hospital, Wuhan University, No.169, Road East lake, Wuhan, 430071 Hubei People’s Republic of China; 2 Department of Nephrology, Tongji Medical College, Wuhan Central Hospital, Huazhong University of Science and Technology, Wuhan, Hubei 430071 People’s Republic of China; 3 Department of Intensive Care Unit, Zhongnan Hospital, Wuhan University, Wuhan, Hubei 430071 People’s Republic of China

**Retraction Note: International Urology and Nephrology (2021) 53:1941–1950** 10.1007/s11255-021-02836-4

The authors have retracted this article due to concerns regarding the integrity of the western blot data presented in Fig. 6. The authors determined that the original data underlying this figure are no longer available. Therefore, this article has been retracted.

All authors agree with this retraction.

